# The Orbitofrontal Cortex Gray Matter Is Associated With the Interaction Between Insomnia and Depression

**DOI:** 10.3389/fpsyt.2018.00651

**Published:** 2018-12-04

**Authors:** Siyi Yu, Zhifu Shen, Rui Lai, Fen Feng, Baojun Guo, Zhengyan Wang, Jie Yang, Youping Hu, Liang Gong

**Affiliations:** ^1^Department of Acupuncture & Tuina, Chengdu University of Traditional Chinese Medicine, Chengdu, China; ^2^Department of Anesthesiology, People's Hospital of Deyang, Deyang, China; ^3^Affiliated Hospital of Chengdu University of Traditional Chinese Medicine, Chengdu, China; ^4^Department of Pain Management, Sichuan Integrative Medicine Hospital, Chengdu, China; ^5^Department of Neurology, Chengdu Second People's Hospital, Chengdu, China; ^6^Department of Neurology, Affiliated ZhongDa Hospital, School of Medicine, Southeast University, Nanjing, China

**Keywords:** insomnia, depression, comorbidity, structural MRI, orbitofrontal cortex

## Abstract

Insomnia and depression are highly comorbid symptoms in both primary insomnia (PI) and major depressive disorder (MDD). In the current study, we aimed at exploring both the homogeneous and heterogeneous brain structure alteration in PI and MDD patients. Sixty-five MDD patients and 67 matched PI patients were recruited and underwent a structural MRI scan. The subjects were sub-divided into four groups, namely MDD patients with higher or lower insomnia, and PI patients with higher or lower severe depression. A general linear model was employed to explore the changes in cortical thickness and volume as a result of depression or insomnia, and their interaction. In addition, partial correlation analysis was conducted to detect the clinical significance of the altered brain structural regions. A main effect of depression on cortical thickness was seen in the superior parietal lobe, middle cingulate cortex, and parahippocampal gyrus, while a main effect of insomnia on cortical thickness was found in the posterior cingulate cortex. Importantly, the interaction between depression and insomnia was associated with decreased gray matter volume in the right orbitofrontal cortex, i.e., patients with co-occurring depression and insomnia showed smaller brain volume in the right orbitofrontal cortex when compared to patients with lower insomnia/depression. These findings highlighted the role of the orbitofrontal cortex in the neuropathology of the comorbidity of insomnia and depression. Our findings provide new insights into the understanding of the brain mechanism underlying comorbidity of insomnia and depression.

## Introduction

Insomnia represents a common symptom seen in the world population (about 30%), with about 6–10% of the adult population reaching the diagnostic criteria for Primary insomnia (PI) (1). In contrast, major depressive disorder (MDD) is the second cause of disability worldwide, which is to become the world's most frequent and economically burdensome illness by 2030 (2, 3). Sleep complaints, especially the symptom of insomnia, are reported in up to 90% of MDD patients and can profoundly impact both the severity of depression and the course of the illness (4, 5). In addition, about 20% of patients with insomnia suffer from depression (6, 7). Furthermore, insomnia was found to be a predictor of depression given that non-depressed people with insomnia have a 2-fold risk to develop depression according to a recent meta-analysis of longitudinal epidemiological studies (8). These findings suggest that the link between insomnia and depression is bidirectional. In addition, both antidepressants and hypnotic medication are commonly prescribed to patients with the combined condition of depression and insomnia (9, 10). Therefore, due to the heterogeneity and homogeneity between insomnia and depression, exploring the common and different brain mechanisms underlying such symptoms may help refine existing depression and insomnia treatments and develop personalized treatment for PI and MDD.

In the last decades, accumulating neuroimaging studies suggested PI and MDD to be associated with some functional and structural alterations in the brain of patients (11, 12). For example, Winkelman et al. found that patients with chronic PI reported an increased cortical volume in the rostral anterior cingulate cortex (rACC) when compared to normal sleepers, which was an indication of clinical severity (13). Other studies also reported volumetric differences in the frontal cortex, OFC, parietal cortex, precuneus and hippocampus (14, 15). In contrast, neuroimaging studies in patients with MDD described smaller volumes of the hippocampus, thalamus, insula, frontal lobe, orbitofrontal cortex and rACC (16, 17). However, due to the heterogeneity between the two diseases, such results regarding the structural brain alteration were not always consistent (11, 18). More recently, an increasing number of researchers pay attention on both the common and different brain mechanisms underlying insomnia and depression. For example, Cheng et al. conducted a study on a bit sample of healthy individuals and found an increased functional connectivity in the lateral orbitofrontal cortex (OFC), dorsolateral prefrontal cortex, anterior and posterior cingulate cortex and insula, which was associated with both sleep and depressive scores (19). Furthermore, Liu et al. reported increased amplitude of low-frequency fluctuations (ALFF) during the resting state in the right inferior frontal gyrus and anterior insula in MDD patient with insomnia when compared to MDD patients without insomnia. In addition, they suggested that the abnormal ALFF was associated with sleep disturbance scores (20). Moreover, Li et al. found that patients with PI had reduced gray matter volume in the middle cingulate cortex, which was significantly associated with self-rating for depression score (21). Yang et al. suggested that decreased gray matter volume both in the left lingual gyrus and cerebellum predicts insomnia in female MDD patients (22). Considering the high comorbidity of insomnia and depression in the two neuropsychiatric disorders, only a few study have investigated their interaction effect on brain structure in both PI and MDD patients.

In the present study, we aimed at exploring the potential brain mechanism underlying the comorbidity of insomnia and depression using structural magnetic resonance imaging (MRI). First, we detected the main and the interaction effects of insomnia and depression on brain cortical thickness and volume in four heterogeneous subgroups of patients, i.e., MDD patients with higher or lower insomnia (MDD-HI or MDD-LI), and PI patients with higher or lower depression (PI-HD or PI-LD). Second, we explored the clinical association between the influenced brain regions in each group. Based on previous neuroimaging finding on PI and MDD (21, 23, 24), we hypothesized that the prefrontal cortex, especially the OFC and the anterior cingulate cortex, would be influenced by the interaction between insomnia and depression.

## Methods and Materials

### Participants

The present study is a preliminary and retrospective research, the enrollment is separately for MDD and PI group. All participants were recruited from the outpatient of department of neurology and psychiatry of the Chengdu University of Traditional Chinese Medicine (CDUTCM). We selected sixty-five MDD patients and 67 age-, gender-, and education-matched PI patients (Table 1). This study was approved by the Research Ethics Committee of CDUTCM and all participants gave written informed consent. The following eligibility criteria were considered for MDD patients: (1) Met the diagnostic criteria for MDD according to the Diagnostic Statistical Manual of Mental Disorder, fourth Edition (DSM-IV); (2) the Hamilton Rating Scale for Depression-17 (HAMD) score was equal or above 17; (3) Naïve to antidepressant medications or a washout period of at least five half-lives of the previously prescribed medicine was undergone (25, 26); (4) Age between 18 and 55; and (5) Age at onset was < 50 years. The following inclusion criteria were considered for PI patients: (1) Met the diagnostic criteria for PI according to the DSM-IV; (2) complaints of difficulty of falling asleep, maintaining sleep or early awakening for at least 3 months; (3) Age between 18 and 55; and (4) Age at onset was < 50 years. The exclusion criteria for all patients included: (1) a history of other major psychiatric disorders or a neurological illness history, except for anxiety in the current state; (2) substance abuse, including caffeine, nicotine, and alcohol (27); (3) any brain lesions found by a T2 MRI scan.

**Table 1:** Demographic and clinical characteristics for all participants.

**Characteristic**	**PI-HD (*n* = 29)**	**PI-LD (*n* = 28)**	**MDD-HI (*n* = 32)**	**MDD-LI (*n* = 28)**	***F/T/χ^2^* value**	***p*-Value**
Age	39.24 ± 10.96	38.42 ± 11.86	39.21 ± 13.81	35.67 ± 10.37	0.592	0.622
Gender (male/female)	10/19	10/18	15/17	10/18	0.217	0.641^†^
Education (years)	12.96 ± 3.98	13.65 ± 3.73	12.09 ± 3.42	12.96 ± 2.83	1.93	0.129
eTIV (ml)	1,484.48 ± 126.93	1,502.34 ± 132.57	1,480.17 ± 146.82	1,485.93 ± 152.39	0.173	0.915
Duration (months)	82.17 ± 82.85	41.10 ± 33.48	78.10 ± 89.12	75.41 ± 106.28	2.78	0.007
PSQI	14.58 ± 2.13	13.52 ± 1.72	–	–	2.25	0.028
HAMD-S	–	–	4.91 ± 0.82	1.78 ± 1.20	11.91	0.000
SDS	61.00 ± 5.50	46.32 ± 3.67	–	–	11.78	0.000
HAMD	–	–	23.20 ± 4.35	16.36 ± 5.72	5.24	0.000
SAS	54.62 ± 6.09	52.65 ± 4.40	–	–	1.53	0.131
HAMA	–	–	17.40 ± 5.95	14.85 ± 5.79	1.67	0.101

† *The p-value was obtained by chi-square test; other p-values were obtained by a two-way T-test or one way analysis of variance. MDD-HI, major depressive disorder with higher insomnia; MDD-LI, major depressive disorder with lower insomnia; PI-HD, primary insomnia with higher depression; PI-LD, primary insomnia with lower depression. PSQI, Pittsburgh Sleep Quality Index; HAMD, Hamilton Rating Scale for Depression; HAMD-S, HAMD sleep subscale; SDS, self-rating depression scale; SAS, self-rating anxiety scale; HAMA, Hamilton Rating Scale for Anxiety*.

### Behavior Assessment and Subgroup Division

All participants underwent both a clinical and a behavioral assessment, while a neuropsychiatric examination was performed by two experienced neurologists (SY and ZS) who reached a consensus diagnosis. The HAMD for depression severity, the Hamilton Rating Scale for Anxiety (HAMA) for anxiety evaluation, and the HAMD sleep subscale (HAMD-S) for insomnia evaluation were used for assessing MDD patients. Following, according to the HAMD-S score, the MDD group was divided into the MDD subgroup with higher insomnia (MDD-HI, HAMD-S score >3) and the MDD subgroup with lower insomnia (MDD-LD, HAMD-S score < 3) (20, 28, 29). Given that 5 MDD patients reported a HAMD-S score equal to 3, they were excluded from the statistical analysis. In contrast, the Pittsburgh Sleep Quality Index (PSQI) for evaluating the insomnia severity (30), the self-rating depression scale (SDS) for depression severity, and the self-rating anxiety scale (SAS) for anxiety severity evaluation were used for assessing PI patients. Following, the PI group was also divided into two subgroups according to the SDS scores, namely the PI subgroup with higher depression (PI-HD, SDS score >55) and the PI subgroup with lower depression (PI-LD, SDS score < 50). Given that 10 patients reported to have a SDS score between 50 and 55, they were not included in the statistical analysis (31).

### Image Data Acquisition and Processing

All participants underwent MRI scanning on the same 3.0-Tesla magnetic resonance scanner (Discovery MR750, General Electric, Milwaukee, WI, USA) equipped with a standard head coil. All participants were instructed not to consume caffeine, alcohol, or any other psychoactive substance in the 48 h prior to the scan. Tight however comfortable foam padding was used to minimize head motion, and earplugs were employed to reduce the scanner noise. Sagittal 3D T1-weighted images were acquired using a brain volume sequence with the following parameters: repetition time (TR) = 8.16 ms, echo time (TE) = 3.18 ms, flip angle (FA) = 7°, field of view (FOV) = 256 × 256 mm^2^, matrix = 256 × 256; slice thickness = 1 mm, no gap; and 188 sagittal slices. During the MRI examination, all subjects were instructed to relax with their eyes closed without falling asleep or thinking about something. All participants were checked for their wakening status following the scan and they all claimed to be awake during the course of the study.

The images were processed using the standard surface-based workflows in the FreeSurfer version 6.0 (http://surfer.nmr.mgh.harvard.edu/fswiki/recon-all/), including motion correction, non-parametric non-uniform intensity correction, intensity normalization, skull strip, automatic subcortical segmentation, white matter segmentation, tessellation, original surface smoothing, inflation, automatic topology fixer, surface registration, and cortical parcellation (32). The resulting surface reconstruction was visually inspected and manually edited in the following trouble shooting step (https://surfer.nmr.mgh.harvard.edu/fswiki/FsTutorial/TroubleshootingDataV6.0) by a single rater, blind to the diagnostic status. The troubleshooting included skull strip errors, segmentation errors, intensity normalization error, pial surface misplacement, and topological defect. The estimated Total Intracranial Volume (eTIV) was extracted as a covariate in a later analysis (33).

### Statistical Analysis

#### Demographic and Behavioral Data

One-way analysis of variance (ANOVA), independent samples *T*-tests and chi-square tests were conducted using the Statistical Package for the Social Sciences version 20.0 (SPSS, Inc., Chicago, IL, USA) to compare the demographic and behavioral data among groups. The Pearson correlation was employed to examine the relationships between HAMD/SDS scores and HAMD-S/PSQI scores. Considering the difference in both the disease duration between the two PI subgroups and in the HAMD score between the two MDD subgroups, the Pearson Correlation analysis was also conducted to investigate the relationships between both disease duration and SDS score in the PI groups and HAMD-S scores in the MDD groups. The significance was set at *p* < 0.05.

#### Structural Imaging

A general linear model (GLM) analysis was employed to explore the effect of depression, insomnia, and their interaction on the reconstructed cortical surface image (mri_glmfit, FreeSurfer), regressed out the effect of gender, age, years of education, and the eTIV. The design matrices were created by a FreeSurfer Group Descriptor File (http://surfer.nmr.mgh.harvard.edu/fswiki/Fsgdf4G1V). For the main effect of depression, we set the MDD-HI, MDD-LI, and PI-HD as depressed patients, PI-LD as non-depressed patients; For the main effect of insomnia, we set the MDD-HI, PI-LD, PI-HD as insomnia patients, MDD-LI as non-insomnia patients; For the interaction of depression and insomnia effect, we set the MDD-HI, PI-HD as the interaction of depression and insomnia. The the contrast matrix is set as the equation below:

$$\begin{array}{l} Y_{i}=\;\beta_{0}+\;\beta_{1}*Gender+\;\beta_{2}*Age+\beta_{3}*Education+\beta_{4}*eTIV\\ \;+{\beta_{5}*Duration+\beta }_{6}*Ins+\beta_{7}*Dep+\beta_{8}*\left(Ins*Dep\right)+\varepsilon \end{array}$$

where Y_*i*_ represents the thickness or volume of each vertex in cortex of the *i*th participant; *β*_0_ is the intercept of the straight-line fitting in the model; *β*_1_, *β*_2_, *β*_3_, *β*_4_*, and* *β*_5_ stand for the main effect of gender, age, education, eTIV volume, and disease duration, respectively, which were discarded as covariates of no interest in the GLM models. *β*_5_ represents the main effect of depression, *β*_6_ is the main effect of insomnia, *β*_7_ describes the interaction effect of depression and insomnia. The error term ε was assumed to have a Gaussian distribution so that no correlation across participants was shown.

After the GLM analyses, a cluster-wise correction for multiple comparison was processed using the Monte Carlo Simulation (mri_glmfit-sim, FreeSurfer, the vertex-wise threshold is *p* < 0.0001, the cluster-wise *p* < 0.05, iteration is 10,000, adjust *p*-values for two hemispheres) (34).

#### The Relationship Between Structural Imaging Features and Behavior

To further detect whether depression and insomnia influenced the brain structural regions associated with clinical characteristics in both the MDD and PI groups, a Partial correlation analysis was conducted, after controlling for the effect of age, gender, years of education, disease duration and eTIV. The significance was set at *p* < 0.05, and the false discovery rate (FDR) correction was used for multiple comparison correction.

## Results

### Demographic Information and Clinical Characteristics

Significant differences in age, gender, years of education and eTIV were not observed between the four groups. As Table 1 illustrates, the disease duration in the PI-LD subgroup is shorter when compared to other groups. In addition, the PSQI score in the PI-LD subgroup is lower than the one in the PI-HD subgroup (*t* = 2.25, *p* = 0.02), while the HAMD score in the MDD-HL subgroup is higher when compared to the one in the MDD-LI subgroup (*t* = 5.24, *p* < 0.001). In contrast, a significant difference in the anxiety symptom was not found between the two PI and MDD groups. In the MDD group, the HAMD score was positively correlated with both the HAMA score (*r* = 0.46, *p* < 0.001), and the HAMD-S score (*r* = 0.57, *p* < 0.001). In concordance, the PSQI score was positively correlated with the SDS score in the PI group (*r* = 0.27, *p* = 0.027). Significant relationships between disease duration, PSQI and SAS scores were not seen in PI groups. An absence of other significant relationships between the clinical characteristics was present.

### Main Effect of Depression on Brain Structure

**Figure 1:**
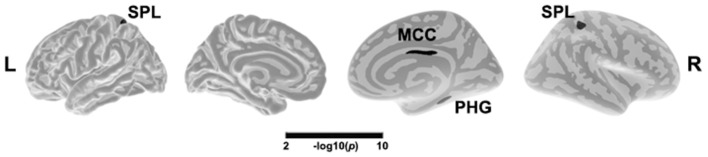
Main effect of depression in cortical thickness across all patients. The color bar indicates the –log10(p) value after clusterwise correction for multiple comparisons using Monte Carlo simulations (vertex *p* < 0.0001, cluster *p* < 0.05). L, left; R, right; MCC, middle cingulate cortex; SPL, superior parietal lobule; PHG, parahippocampal gyrus.

As illustrated in Figure 1 and Table 2, a main effect of depression on cortical thickness was seen in the bilateral superior parietal lobule (left SPL, 2.39 ± 0.16 vs. 2.45 ± 0.10; right SPL, 2.37 ± 0.12 vs. 2.43 ± 0.11), the right middle cingulate cortex (MCC, 2.38 ± 0.15 vs. 2.46 ± 0.12), and the right parahippocampal gyrus (2.42 ± 0.18 vs. 2.45 ± 0.16), after adjusting for the effects of gender, age, and years of education and TIV. The cortical regions affected by depression were thinner in patients with depression when compared to patients with an absence of severe depression symptom. In contrast, a significant effect of depression on cortical volume was not found.

**Table 2:** The depression and insomnia and their interactive effects on cortical thickness and volume.

**Clusters**	**Max *T* value**	**VtxMax**	**TalX**	**TalY**	**TalZ**	**CWP**	**NVtxs**
**MAIN EFFECT OF DEPRESSION ON CORTICAL THICKNESS**							
1. Left superior parietal lobe	7.419	133,615	−16.9	−50.6	61.6	0.0058	292
2. Right superior parietal lobe	5.136	14,752	28.8	−39.5	490.0	0.0006	563
3. Right middle cingulate cortex	5.948	35,246	3.3	−7.3	29.5	0.0020	357
4. Right parahippocampal gyrus	5.825	113,996	35.8	−35.0	−13.8	0.0020	341
**MAIN EFFECT OF INSOMNIA ON CORTICAL THICKNESS**							
1. Right posterior cingulate cortex	6.709	24,061	5.8	−38.4	24.3	0.0026	346
INTERACTIVE EFFECT OF DEPRESSIVE AND INSOMNIA IN CORTICAL VOLUME^*^							
1. Right orbital frontal cortex	3.615	139,780	6.9	0.0	64.8	0.0002	3,508

* *The threshold of multiple comparison correction was set as vertex p at 0.05, and cluster p at 0.05. TalX,Y,Z, the Talairach coordinate of peak vertex; VtxMax, number of peak vertex of the significant cluster; CWP, cluster-wise probability and the nominal p-value; NVtxs, number of vertices in cluster*.

### Main Effect of Insomnia on Brain Structure

**Figure 2:**
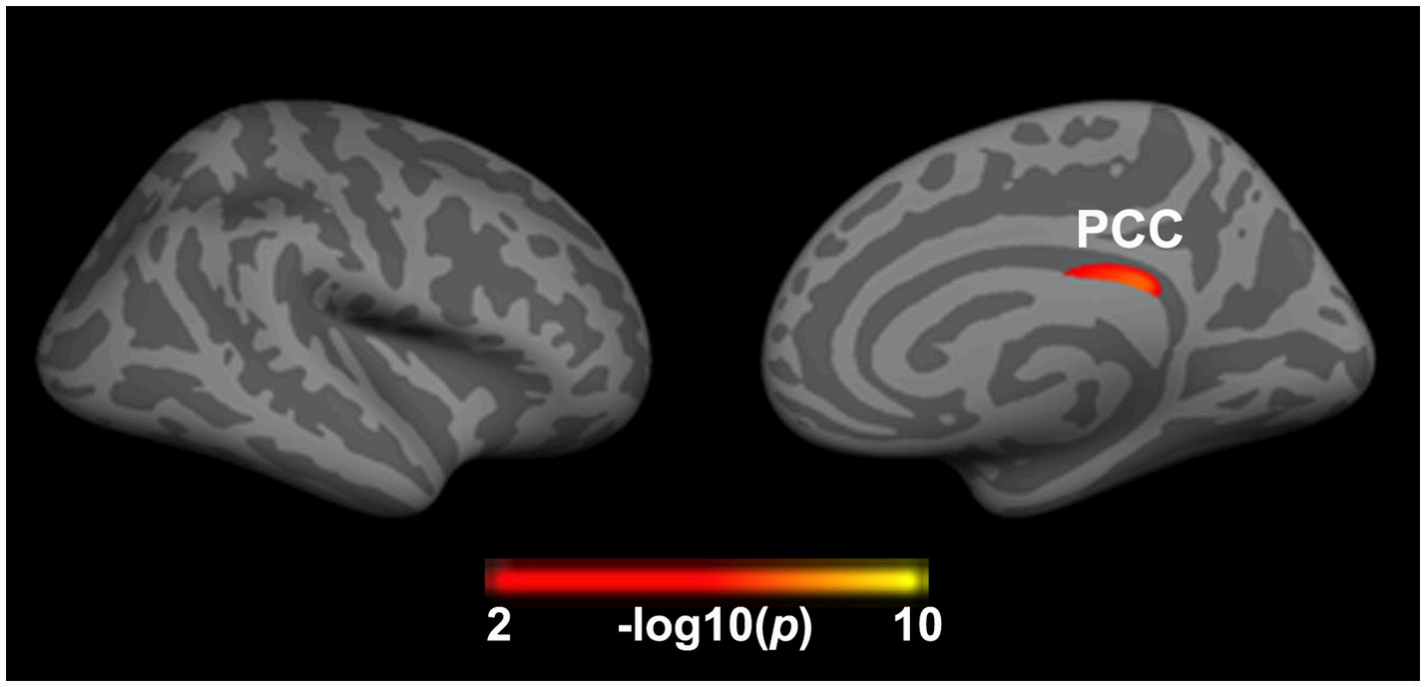
Significant effect of insomnia on right cortical thickness across all patients. The color bar indicates the –log10(p) value after clusterwise correction for multiple comparisons using Monte Carlo simulations (vertex *p* < 0.0001, cluster *p* < 0.05). PCC, posterior cingulate cortex.

After adjusting for the effects of gender, age, and years of education, a main effect of insomnia on cortical thickness was observed in the right PCC (2.39 ± 0.16 vs. 2.43 ± 0.14). As showed in Figure 2 and Table 2, the right PCC was thinner in patients with insomnia when compared to patients with an absence of severe insomnia symptom. In contrast, a significant effect of insomnia on cortical volume was not seen.

### Interaction Effect of Depression and Insomnia on Brain Structure

**Figure 3:**
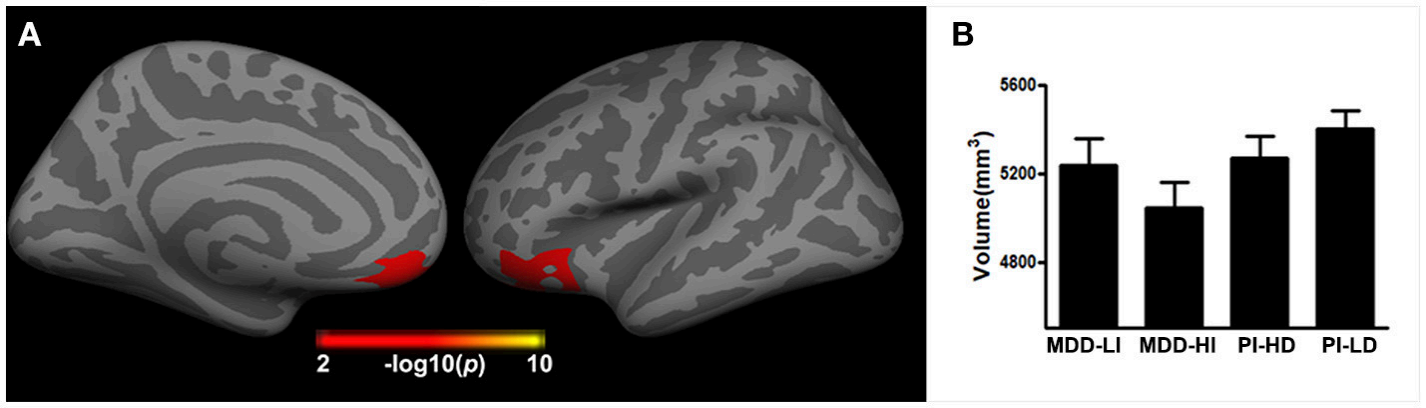
The interactive effect of depression and insomnia on right cortical volume across all patients. **(A)** The interactive effect was found in right orbital frontal cortex; **(B)** The histogram indicated that the joint depression and insomnia showed smaller brain volume in the right orbital frontal cortex.

A significant interaction effect of depression and insomnia on the right orbital frontal cortex (OFC) volume was detected, after both a flexible multiple comparison correction (*p* < 0.05) and controlling for the effects of gender, age, and years of education. As Figure 3 displays, patients with co-occurring depression and insomnia showed smaller brain volume in the right OFC when compared to patients without severe insomnia/depression, in both the MDD and the PI groups. A significant interaction effect of depression and insomnia on cortical thickness was not present after multiple comparison correction.

### Correlation Analysis

An absence of significant FDR-adjusted correlations between influenced brain regions and relative clinical traits in the MDD and PI groups was reported.

## Discussion

To our knowledge, the present study was the first attempt to explore the interaction of insomnia and depression on brain structure in both PI and MDD patients. Three major findings were reported. First, insomnia and depression symptoms were closely associated in both the PI and MDD patients groups. Second, depression influences the brain structure in the SPL, MCC, and parahippocampal gyrus, while insomnia mainly influences the PCC thickness. Third, depression and insomnia interaction contributes to cortical volume loss in the right OFC. These findings indicated that the OFC might be a core region for the neuropathological alteration in comorbidity of insomnia and depression. Therefore, our findings provide new insights into the understanding of the brain mechanism underlying comorbidity of insomnia and depression.

The clinical features of the four subgroups verified the close association and the comorbidity between MDD and PI. In fact, patients with insomnia in the MDD group showed more severe depression, which is consistent with a previous epidemiologic study (35). In concordance, PI patients with depression have a longer disease duration and more severe insomnia, which is positively associated with depression but not with the anxiety symptom. These results were proved also consistent with previous epidemiologic results on the long-term consequences of insomnia (1). The clinical results presented here support the notion that the relationship between insomnia and depression is bidirectional, and this relationship can change over time following disease progression or release (7).

In the present study, we investigated the insomnia and depression effects on brain structure in a pooled patient group. This approach enables, in fact, the detection of the insomnia/depression effect in two patient groups and might reveal the common neurobiology underlying such symptoms. Only cortical thickness was significantly influenced by insomnia and depression in the pooled population. Patients with depression showed thinning of the SPL, MCC, and parahippocampal gyrus. The SPL is involved in spatial cognition and sensorimotor integration (36, 37); previous studies also reported decreased gray matter volume and reduced functional connectivity in the SPL in MDD patients (38, 39). Although the alteration of the MCC is less commonly reported in depression studies, the posterior MCC is involved in attentional control during emotion regulation and showed a functional abnormality in MDD patients (40, 41). The present result provides a new insight into emotion regulation abnormality in patient with depression. Last, the parahippocampal gyrus is important for episodic memory and emotion cues processing (42); the thinning parahippocampal gyrus here found is consistent with previous structural imaging results in MDD patients (43), and indicated a common structural basis underlying both emotion and episodic memory processing in the patients with depression. Regarding patients with insomnia, the common structural alteration was seen in PCC, which is the core region of the default mode network and has been considered to be involved in emotion, memory and intrinsic control, and to be represent a neural substrate for human awareness (44, 45). The metabolism, functional connectivity abnormality and structural connectivity of the anterior default mode network was also reported in PI patients (46, 47). Generally, our findings on the main effect of insomnia and depression highlighted the morphological alteration in the thickness of the SPL, MCC, and parahippocampal gyrus in depression and of the PCC in insomnia.

Our most important finding was the localization of the insomnia/depression interaction effect on brain structure in the right OFC. The orbitofrontal area is involved in emotion, especially in reward processing, decision-making, and problem-solving abilities (48, 49). Previously, gray matter decrements in the OFC were reported in insomnia and depression patients, separately (50, 51). The decreased PFC volume may explain the insufficient decision-making and problem-solving abilities, and the attenuated recognition of environmental temperature in insomnia patients (24, 52). In addition, the reduced OFC volume may account for the distorted emotional stimuli evaluation, abnormal emotional and visceral regulation, anhedonia symptoms as well (23, 50). Interestingly, we found that co-occurrence of insomnia and depression is related to the worst volume decrease seen in the orbitofrontal cortex, especially, in MDD patients with insomnia. Baglioni and Riemann proposed a hypothesis stating that the link between persistent insomnia and depression is the alteration of the arousal system and its subsequent impact on affective and cognitive systems (53). Given that OFC receives widespread projections from arousal systems, including thalamus and amygdala (54), the decreased OFC volume may indicate an abnormal top-down control mechanism for arousal systems and dysfunctional emotional regulation and reward processing in patients with comorbidity of insomnia and depression. Overall, our results suggested that OFC is an essential area underlying the neuropathological mechanism of the comorbidity of insomnia and depression.

Two factors are likely to account for the absence in clinical significance of the alteration in cortical morphology of regions. First, considering that the behavioral assessments of depression and insomnia are not conformed between groups, we can only conduct the correlation analysis separately. Second, given that the clinical characteristics were robustly associated with demographic factors and relative stability of brain structure, some recent studies found that cortical structure feature is a relatively weak discriminant factor for current episode clinical status (55, 56). Further studies should include detailed and conformed neuropsychological tests, and a larger sample to verify this.

The limitations of the current study will now be highlighted. First, the present study is a preliminary and retrospective research, so the present study did not use the same estimating tool for depression and insomnia, we cannot explore the relationship between the influenced structural region and the clinical traits in a pooled group. In addition, the HAMD subscale of insomnia scores only has three items to estimate the insomnia, the HAMD total score is positively correlated with HAMD-S score, so it's difficult to completely remove the depression severity effect in the data analysis. These limitations regarding the clinical evaluation restricted the elaboration of our findings on brain structure. Second, not only insomnia, but also hypersomnia, is present in MDD patients (5); therefore, future studies should explore the brain mechanism underlying hypersomnia in MDD patients. Third, significant differences were found between the PI-HD and PI-LD groups in the PSQI total score, and between the MDD-HI and MDD-LI groups in the HAMD total score, which might bias the findings to uncertain extent. Fourth, the present study strictly focused on the structural change of cortical regions in depression and insomnia, whereas the subcortical and cerebellar regions were unexplored. Future studies should explore these issues further. Fifth, the present study did not include a healthy control group, which again limits the discussion of our findings. Lastly, considering that our study had a cross-sectional design with relatively small sample sizes, future research using larger sample sizes, data pooling, and longitudinal designs is deemed necessary.

In conclusion, our present results indicated that the OFC may be a core region for the neuropathological mechanism in comorbidity of insomnia and depression. In addition, our findings provide new insights into the understanding of the brain mechanism underlying comorbidity of insomnia and depression and the development of rational treatment strategies for these patients.

## Author Contributions

SY, YH, and LG contributed to the conception and design of study. RL, BG, ZW, and FF contributed to the clinical estimate acquisition of imaging data. SY and LG performed imaging data analysis. SY and ZS wrote the manuscript. ZS and JY contributed revising the manuscript logically for important theoretical content. All authors contributed to the manuscript and have approved the final manuscript.

### Conflict of Interest Statement

The authors declare that the research was conducted in the absence of any commercial or financial relationships that could be construed as a potential conflict of interest.
